# Population-level effects of the national diabetes prevention programme (FIN-D2D) on the body weight, the waist circumference, and the prevalence of obesity

**DOI:** 10.1186/1471-2458-11-350

**Published:** 2011-05-19

**Authors:** Titta M Salopuro, Timo Saaristo, Heikki Oksa, Hannu Puolijoki, Mauno Vanhala, Tapani Ebeling, Leo Niskanen, Jaakko Tuomilehto, Matti Uusitupa, Markku Peltonen

**Affiliations:** 1 Pirkanmaa Hospital District, Tampere, Finland; 2 Diabetes Prevention Unit, Department of Chronic Disease Prevention, National Institute for Health and Welfare, Helsinki, Finland; 3 Finnish Diabetes Association, Tampere, Finland; 4 South Ostrobothnia Hospital District, Seinäjoki, Finland; 5 Unit of Family Practice, Central Finland Hospital District, Jyväskylä, Finland; 6 Institute of Public Health and Clinical Nutrition, University of Eastern Finland, Kuopio, Finland; 7 Unit of Primary Health Care, Kuopio University Hospital, Kuopio, Finland; 8 Oulu University Hospital, Northern Ostrobothnia Hospital District, Oulu, Finland; 9 Department of Medicine, Kuopio University Hospital, Hospital District of Northern Savo, Kuopio, Finland; 10 Department of Public Health, University of Helsinki, Helsinki, Finland; 11 Research Unit, Kuopio University Hospital, Kuopio, Finland

## Abstract

**Background:**

The implementation project of the national diabetes prevention programme in Finland, FIN-D2D, was carried out in primary health care in the area of five hospital districts during 2003-2007.

**Methods:**

The population strategy of FIN-D2D was primarily aimed at increasing the awareness of type 2 diabetes and preventing obesity. To investigate the effects of this strategy, we studied the changes in the prevalence of obesity, overweight, and central obesity among a random independent sample of individuals aged 45-74 years in the FIN-D2D area; and assessed whether they differed from a sample of individuals in the control area, which consisted of four geographical areas not participating in FIN-D2D (FINRISK study). Data was obtained for 5850/ 6406 (in the beginning/ in the end) individuals. The duration of the observation period varied from three to five years.

**Results:**

The mean body weight decreased from 78.7 to 78.1 kg (*p = *0.041) in the FIN-D2D area, and from 78.7 to 78.0 kg (*p = *NS) in the control area. The prevalence of obesity (BMI ≥30 kg/m^2^) decreased in the FIN-D2D area (26.5% vs. 24.4%, *p = *0.015), and in the control area (28.4% vs. 25.2%, *p = *0.005). The prevalence of morbid obesity (BMI ≥40 kg/m^2^) remained unchanged in the FIN-D2D area, but increased in the control area (1.2% vs. 2.3%, *p = *0.007). The mean waist circumference remained unchanged in the FIN-D2D area, but increased in the control area (92.8 vs. 94.0 cm, *p = *0.005).

**Conclusions:**

The prevalence of obesity may be decreasing among 45-74 year old Finns. We still need a longer time perspective and future studies to see whether this favourable trend can be sustained in Finland. The actions of this implementation project can at least partly explain the differences in the mean waist circumference and the prevalence of morbid obesity between the intervention and control areas.

## Background

During the last decades, the prevalence of obesity has doubled in adults in the United States [1] and in Finland [2]. However, recent reports have shown that the obesity epidemic might be slowing down [3,4]. Obesity is strongly associated with a reduction in life expectancy, as well as with an increased risk of type 2 diabetes (T2DM), coronary heart disease, and certain cancers [5]. Furthermore, obesity increases the risk of musculoskeletal disorders [6] and early retirement [7]. Obesity increases the risk of T2DM to a much greater extent than it increases the risk of other diseases, independently of age, race, and physical activity [8]. Furthermore, numerous large-scale studies have shown that overall mortality rises steadily as a function of increasing body weight [9], and even small increases in weight across a population can have a devastating impact on public health.

According to a large meta-analysis, more than a half of the Europeans will suffer from hyperglycemia and T2DM during their lifetime [10,11]. Thus, the economic burden is already big and will be huge in the forthcoming years [12]. The most efficient way to manage T2DM and its complications is to prevent the onset of diabetes and prediabetic states. As several intervention trials have demonstrated, prevention of T2DM and its complications is possible and efficient by modest changes in lifestyle, primarily in diet and physical activity [13-17]. Moreover, it has been shown that the achieved benefit from lifestyle interventions can be sustained for years after the active period [18-20]. A recent meta-analysis [21] has shown that lifestyle interventions for patients at high risk of diabetes, delivered by a variety of healthcare providers in routine clinical settings, are feasible but appear to be of limited clinical benefit one year after intervention. Although weight and waist circumference reductions were achieved, translation into routine practice had less effect on diabetes risk reduction [21].

To translate the scientific evidence regarding prevention of T2DM into daily practice and public health, the first large-scale national type 2 diabetes prevention programme was carried out in Finland, covering three concurrent strategies: the population strategy, the high-risk strategy, and the strategy of early diagnosis and treatment [22,23]. The baseline results of the implementation project, FIN-D2D, have shown that only 58% of men and 67% of women have normal glucose tolerance, and the disturbances of glucose metabolism are closely associated with obesity and central obesity [24]. FIN-D2D supported the population strategy that aimed to improve the health of the entire population through a healthy diet and regular physical activity [22]. Different manners, such as media communication, training, lifestyle counselling and an extensive network supporting these activities, were used to prevent obesity, metabolic syndrome and other risk factors of T2DM [25]. The project was carried out in five hospital districts in Finland in 2003-2007.

In this study based on two independent surveys with a follow-up of 3 to 5 years, we examined the effects of FIN-D2D on the prevalence of obesity, overweight, and central obesity at the population-level, among 45-74 years old Finnish men and women, and assessed whether these effects differ from the respective changes among Finnish population living in four geographical areas not participating in FIN-D2D. The results of this study reflect the achievements of all three concurrent strategies at the population-level, whereas the 1-year results of the high-risk strategy have been published elsewhere [26].

## Methods

FIN-D2D was carried out in five hospital districts, covering altogether 1.5 million people (South Ostrobothnia, Central Finland, Pirkanmaa, Northern Ostrobothnia, and Northern Savo) during 2003-2007. As a part of the evaluation, population-based health examination surveys with independent random samples from the population were conducted in the beginning and at the end of the project period. Individuals aged 45-74 years, stratified according to sex, 10-year age groups (45-54, 55-64, and 65-74 years) and five geographical areas, were selected from the National Population Register at the beginning of the project (year 2002 for Northern Ostrobothnia and Northern Savo, and year 2004 for South Ostrobothnia, Central Finland, and Pirkanmaa) and at the end of the project (year 2007 for all five areas). Data on anthropometric measurements were obtained for 3812 and 4156 individuals (at the beginning and at the end, respectively).

**Figure 1:**
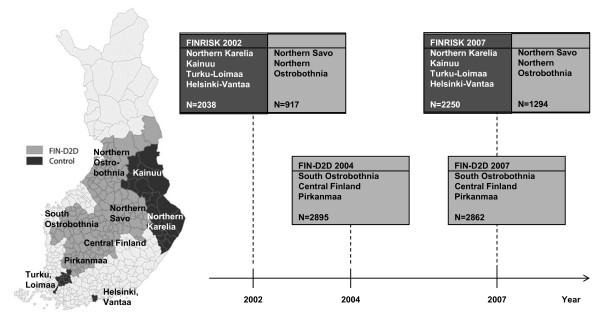
**Formation of the study population**. The FIN-D2D area covers the hospital districts of South Ostrobothnia, Central Finland, Pirkanmaa, Northern Ostrobothnia, and Northern Savo (light grey colour on the map and in the boxes), and the control area covers Northern Karelia, Kainuu, Turku and Loimaa region, and the cities of Helsinki and Vantaa (dark grey colour on the map and in the boxes)

The FINRISK study [27,28] is a chronic disease risk factor survey of the middle-aged Finnish population that has been carried out every five years since 1972. The geographical FINRISK study areas which were not participating in FIN-D2D project were used as a control area in this study (Figure 1). In this study we used the data from FINRISK-2002 and FINRISK-2007 studies covering the following geographical areas in Finland: Northern Savo, Northern Karelia, Northern Ostrobothnia, Kainuu, Turku and Loimaa region, and the cities of Helsinki and Vantaa. Northern Savo and Northern Ostrobothnia belong to the FIN-D2D area (hospital districts of Northern Savo and Northern Ostrobothnia, respectively), thus the data from these areas were combined with the FIN-D2D data from the hospital districts of South Ostrobothnia, Central Finland, and Pirkanmaa, to constitute the FIN-D2D area [25]. Study participants aged 45-74 years were included in this study. Data on anthropometric measurements were obtained for 2038 and 2250 (years 2002 and 2007, respectively) individuals from the areas of Northern Karelia, Kainuu, Turku-Loimaa, and Helsinki-Vantaa [25]. This study population will be referred to as the control area in this study.

The primary strategy of the FIN-D2D was a high-risk strategy, the aim of which was to include prevention of diabetes and reduction of cardiovascular risk factor levels among high-risk individuals in daily routines in primary health care centers and occupational health care outpatient clinics [26]. The population strategy focused on raising awareness of diabetes and its risk factors in the overall population, and the early treatment strategy focused on care of diabetes in individuals who had screening-detected diabetes [26]. The different strategies have been described in detail elsewhere [23,26]. Subjects were invited by mail to a clinical examination. Together with the invitation, they also received a self-administered questionnaire on socioeconomic background, medical history, and health behaviour. They were asked to complete the questionnaire at home, and bring it with them to the health examination, which was carried out according to the WHO MONICA project protocol [29]. At the study site, height, weight and waist circumference were measured using a standardized protocol. Height was measured to the nearest 0.1 cm. Body weight of the participants wearing usual light indoor clothing without shoes was measured with a 0.1 kg precision on a balanced beam scale (FINRISK, FIN-D2D 2004) or on an electronic scale (FIN-D2D 2007).

BMI was calculated as weight (kg) divided by height squared (m^2^). Overweight and obesity were defined as BMI 25-29.9 kg/m^2 ^and ≥30 kg/m^2^, respectively. Obesity was further divided into class 1 (BMI 30-34.9), class 2 (BMI 35-39.9), and class 3 (BMI ≥40) obesity [25]. Central obesity was defined according to the IDF criteria [30]: waist circumference ≥94 cm in men, and ≥80 cm in women, and according to the criteria used by WHO [31]: waist circumference ≥102 cm in men, and ≥88 cm in women.

Statistical analysis was done by the SPSS program version 17 (SPSS, Chigago, IL, USA). The univariate ANOVA was used to test the difference in the continuous variables and in the group-specific changes in them. Logistic regression analysis was performed to evaluate the differences in various prevalences between the study areas. Interaction term 'study area × time point' was used to assess differences in changes between the areas. Adjustment for age and gender was done, when appropriate. In the stratified analysis, genders and three age groups (45-54, 55-64, and 65-74 years) were analysed separately. Continuous variables are presented as mean (standard deviation; SD) and categorical variables as percentages.

The study protocols for FIN-D2D population survey study as well as the FINRISK study were approved by the Ethical Committee of the Hospital District of Helsinki and Uusimaa. All the surveys were conducted according to the ethical rules of the National Public Health Institute, and the investigations were performed in accordance with the Declaration of Helsinki. All participants gave their written informed consent prior to participation in the study.

## Results

The overall response rate was 60% and 63% (beginning and end, respectively) in the FIN-D2D area, and 49% and 58% (beginning and end, respectively) in the control area (Table 1). The mean age of the study participants was higher at the end than at the beginning in both study populations, due to the fact that the oldest age group (65-74 years) was not included in the following FINRISK-2002 study areas: Turku and Loimaa region, Northern Savo, Northern Ostrobothnia and the Kainuu area. No differences in age were seen between the FIN-D2D and control areas at either time point (Table 1).

**Table 1:** Characteristics of the study participants according to sex, study area and timepoint

	FIN-D2D area		Control area	
	**Beginning**	**End**	**Beginning**	**End**

**Men**				
Number of subjects, invited	3175	3329	2060	1921
Participated (%)	1789 (56.3)	1982 (59.5)	984 (47.8)	1066 (55.5)
Age, years	59.1 (8.1)	60.1 (8.5)*	59.3 (7.8)	60.2 (8.2)*
Weight, kg	85.6 (14.2)	84.4 (14.3)*	85.1 (13.2)	84.5 (14.4)
BMI, kg/m^2^	27.8 (4.1)	27.4 (4.2)*	27.9 (3.9)	27.6 (4.3)
Waist, cm	99.2 (11.6)	99.2 (11.8)	98.5 (11.1)	99.1 (12.1)
**Women**				
Number of subjects, invited	3173	3319	2067	1931
Participated (%)	2023 (63.8)	2174 (65.5)	1054 (51.0)	1184 (61.3)
Age, years	58.5 (8.2)	59.3 (8.3)*	58.3 (7.8)	59.7 (8.4)*
Weight, kg	72.6 (13.7)	72.3 (14.2)	72.7 (13.7)	72.1 (14.2)
BMI, kg/m^2^	27.7 (5.1)	27.6 (5.3)	28.0 (5.0)	27.6 (5.4)*
Waist, cm	89.2 (13.2)	89.7 (13.3)	87.5 (12.6)	89.4 (13.5)*
**Total**				
Number of subjects, invited	6348	6648	4127	3852
Participated (%)	3812 (60.1)	4156 (62.5)	2038 (49.4)	2250 (58.4)
Age, years	58.8 (8.1)	59.7 (8.4)*	58.8 (7.8)	60.0 (8.3)*
Weight, kg	78.7 (15.4)	78.1 (15.5)*	78.7 (14.8)	78.0 (15.6)
BMI, kg/m^2^	27.8 (4.7)	27.5 (4.8)*	27.9 (4.5)	27.6 (4.9)*
Waist, cm	93.9 (13.4)	94.3 (13.5)	92.8 (13.1)	94.0 (13.7)*

Data are presented as means (standard deviation) except where noted otherwise.

* *p*< 0.05 (adjusted for age) for the change from the beginning to the end of the study.

### Changes in body weight and BMI

The mean body weight decreased significantly only in men in the FIN-D2D area (from 85.6 ± 14.2 to 84.4 ± 14.3 kg, *p = *0.030) (Table 1). When stratified into age groups, no significant changes were seen, although similar decreasing trend was observed in nearly all age groups in both genders. Among men, weight seemed to decrease with increasing age. The mean BMI decreased significantly only in men in the FIN-D2D area and women in the control area (Table 1). When stratified into age groups, BMI showed a decreasing trend in both genders, areas, and all age groups across the studies, but most so among the youngest men in the FIN-D2D area (27.6 ± 4.2 vs. 27.0 ± 4.2 kg/m^2^, *p = *0.015). Among women, BMI increased with increasing age.

### Changes in the prevalence of obesity

**Figure 2:**
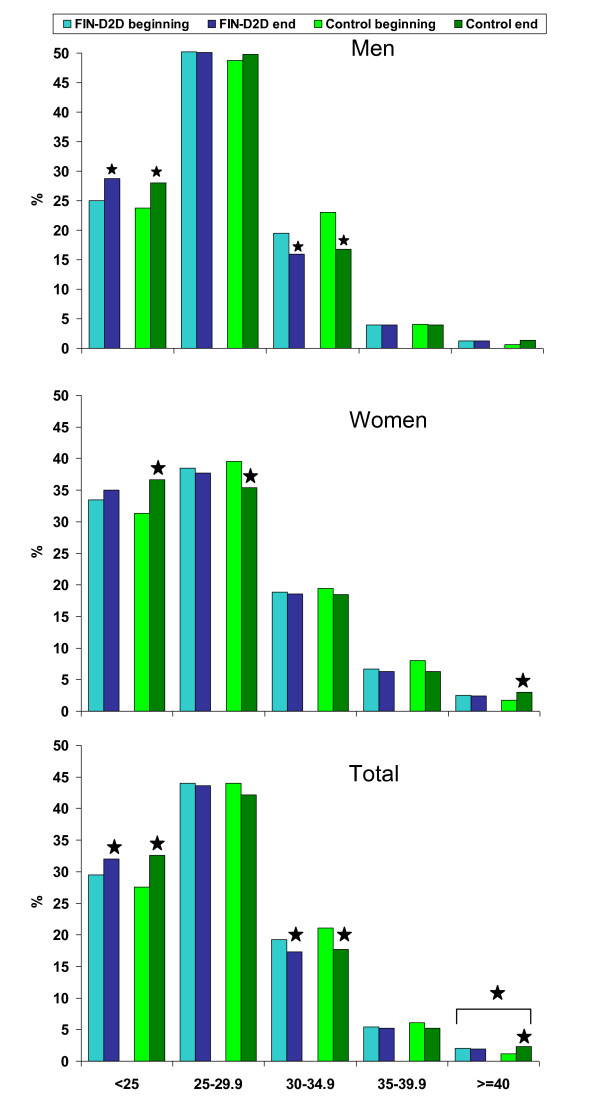
**Categories of BMI**. Changes in the prevalence of normal weight (BMI < 25 kg/m^2^), overweight (BMI 25-29.9 kg/m^2^), obesity class 1 (BMI 30-34.9 kg/m^2^), class 2 (BMI 35-39.9 kg/m^2^), and class 3 (BMI ≥40 kg/m^2^) are shown by sex and study area. Significant (*p <*0.05) age-adjusted changes and interactions for study area × time point are marked (⋆)

The prevalence of obesity was significantly lower in the 2007 survey among men in both study areas (Table 2). Respectively, the prevalence of normal weight was higher in the 2007 survey, especially among the youngest age group in men. The prevalence of normal weight was higher and the prevalence of overweight was lower in the 2007 survey among women in the control area (Table 3), whereas no changes among women in the FIN-D2D area were observed. The prevalence of obesity increased with increasing age among women (Table 3). Altogether, normal weight was more prevalent among women than among men (34.2% vs. 26.6%), whereas overweight was more prevalent among men than among women (49.8% vs. 37.8%) (Figure 2).

**Table 2:** Age-adjusted prevalence (95% confidence interval, CI) of normal weight, overweight, obesity and central obesity in the beginning of the study and the changes in them in men according to the study area and age group

	FIN-D2D area					Control area					
									
	%	(95% CI)	Change	(95% CI)	p*	%	(95% CI)	Change	(95% CI)	p*	p**
**45-54 years**											
Number of subjects	587		581			288		302			
Normal weight	29.1	(25.4 - 32.8)	6.2	(0.8 - 11.5)	.027	28.1	(22.9 - 33.3)	5.0	(-2.5 - 12.4)	.185	.830
Overweight	47.5	(43.5 - 51.6)	-1.1	(-6.8 - 4.7)	.667	46.2	(40.4 - 52.0)	1.8	(-6.3 - 9.9)	.769	.561
Obesity	23.3	(19.9 - 26.8)	-5.1	(-9.8 - -0.4)	.043	25.7	(20.6 - 30.8)	-6.8	(-13.5 - -0.1)	.066	.720
Class 1 obesity	18.1	(14.9 - 21.2)	-4.8	(-9.0 - -0.6)	.033	22.9	(18.0 - 27.8)	-8.3	(-14.6 - -2.1)	.014	.474
Class 2 obesity	3.6	(2.1 - 5.1)	-0.1	(-2.3 - 2.0)	.934	2.1	(0.4 - 3.7)	0.9	(-1.7 - 3.4)	.453	.514
Class 3 obesity	1.7	(0.7 - 2.8)	-0.2	(-1.6 - 1.3)	.813	0.7	(-0.3 - 1.7)	0.6	(-1.0 - 2.3)	.457	.447
Central obesity IDF	60.5	(56.5 - 64.4)	-3.2	(-8.8 - 2.5)	.310	56.6	(50.8 - 62.4)	-1.0	(-9.0 - 7.1)	.899	.659
Central obesity WHO	32.0	(28.2 - 35.8)	-2.8	(-8.1 - 2.5)	.379	29.9	(24.5 - 35.2)	-3.4	(-10.7 - 3.9)	.431	.862
**55-64 years**											
Number of subjects	716		657			422		364			
Normal weight	22.2	(19.2 - 25.3)	4.6	(0.0 - 9.1)	.050	21.8	(17.8 - 25.8)	4.0	(-1.9 - 10.0)	.174	.930
Overweight	52.0	(48.3 - 55.6)	-0.5	(-5.8 - 4.8)	.780	50.0	(45.2 - 54.8)	-0.8	(-7.9 - 6.2)	.782	.960
Obesity	25.8	(22.6 - 29.1)	-4.1	(-8.6 - 0.4)	.099	28.2	(23.9 - 32.5)	-3.2	(-9.4 - 3.0)	.320	.814
Class 1 obesity	19.7	(16.8 - 22.6)	-3.9	(-7.9 - 0.2)	.086	22.5	(18.5 - 26.5)	-3.8	(-9.5 - 1.9)	.191	.959
Class 2 obesity	4.9	(3.3 - 6.5)	-0.9	(-3.1 - 1.3)	.363	5.0	(2.9 - 7.1)	-0.9	(-3.8 - 2.1)	.569	.920
Class 3 obesity	1.3	(0.4 - 2.1)	0.7	(-0.6 - 2.1)	.258	0.7	(-0.1 - 1.5)	1.5	(-0.2 - 3.1)	.074	.414
Central obesity IDF	70.6	(67.3 - 74.0)	-0.9	(-5.7 - 4.0)	.749	67.5	(63.0 - 72.0)	4.1	(-2.4 - 10.6)	.231	.251
Central obesity WHO	37.9	(34.3 - 41.5)	3.0	(-2.2 - 8.2)	.217	37.9	(33.3 - 42.6)	2.6	(-4.3 - 9.4)	.473	.863
**65-74 years**											
Number of subjects	486		744			274		400			
Normal weight	24.1	(20.3 - 27.9)	1.2	(-3.8 - 6.1)	.588	21.9	(17.0 - 26.8)	4.1	(-2.5 - 10.7)	.227	.489
Overweight	50.8	(46.4 - 55.3)	0.8	(-4.9 - 6.5)	.828	49.3	(43.3 - 55.2)	2.5	(-5.2 - 10.2)	.515	.726
Obesity	25.1	(21.2 - 29.0)	-2.0	(-6.9 - 2.9)	.422	28.8	(23.4 - 34.2)	-6.6	(-13.2 - 0.1)	.051	.291
Class 1 obesity	20.8	(17.2 - 24.4)	-2.6	(-7.1 - 1.9)	.276	23.7	(18.7 - 28.8)	-7.0	(-13.1 - -0.9)	.023	.273
Class 2 obesity	3.3	(1.7 - 4.9)	1.1	(-1.1 - 3.4)	.370	4.7	(2.2 - 7.3)	0.0	(-3.3 - 3.3)	.969	.529
Class 3 obesity	1.0	(0.1 - 1.9)	-0.5	(-1.5 - 0.5)	.297	0.4	(-0.4 - 1.1)	0.4	(-0.8 - 1.6)	.538	.296
Central obesity IDF	74.1	(70.2 - 78.0)	-2.1	(-7.2 - 3.0)	.385	69.3	(63.8 - 74.8)	-0.5	(-7.6 - 6.6)	.887	.690
Central obesity WHO	39.9	(35.5 - 44.3)	0.1	(-5.5 - 5.7)	.976	38.3	(32.5 - 44.1)	1.4	(-6.2 - 8.9)	.730	.787
**Total**											
Number of subjects	1789		1982			984		1066			
Normal weight	25.0	(23.0 - 27.0)	4.1	(1.2 - 6.9)	.005	23.7	(21.0 - 26.3)	4.6	(0.8 - 8.4)	.018	.791
Overweight	50.2	(47.9 - 52.5)	-0.3	(-3.5 - 2.9)	.866	48.7	(45.6 - 51.8)	1.0	(-3.3 - 5.3)	.654	.640
Obesity	24.8	(22.8 - 26.8)	-3.8	(-6.5 - -1.1)	.006	27.6	(24.8 - 30.4)	-5.6	(-9.3 - -1.8)	.004	.496
Class 1 obesity	19.5	(17.6 - 21.3)	-3.8	(-6.2 - -1.3)	.003	23.0	(20.3 - 25.6)	-6.3	(-9.7 - -2.8)	.000	.291
Class 2 obesity	4.0	(3.1 - 4.9)	0.0	(-1.3 - 1.2)	.949	4.1	(2.8 - 5.3)	-0.1	(-1.9 - 1.6)	.867	.994
Class 3 obesity	1.3	(0.8 - 1.9)	0.0	(-0.7 - 0.7)	.976	0.6	(0.1 - 1.1)	0.8	(0.0 - 1.7)	.063	.124
Central obesity IDF	68.2	(66.1 - 70.4)	-1.9	(-4.9 - 1.0)	.204	64.8	(61.8 - 67.8)	0.6	(-3.5 - 4.7)	.776	.325
Central obesity WHO	36.5	(34.3 - 38.8)	0.2	(-2.9 - 3.2)	.919	35.7	(32.7 - 38.7)	0.0	(-4.1 - 4.2)	.986	.982

*p-value for change, adjusted for age

**Interaction for study area × timepoint, adjusted for age

**Table 3:** Age-adjusted prevalence (95% confidence interval, CI) of normal weight, overweight, obesity and central obesity in the beginning of the study and the changes in them in women according to the study area and age group

	FIN-D2D area					Control area					
									
	%	(95% CI)	Change	(95% CI)	p*	%	(95% CI)	Change	(95% CI)	p*	p**
**45-54 years**											
Number of subjects	725		713			369		358			
Normal weight	41.5	(37.9 - 45.1)	2.1	(-3.0 - 7.2)	.489	40.1	(35.1 - 45.1)	7.4	(0.2 - 14.6)	.067	.270
Overweight	35.2	(31.7 - 38.7)	-1.4	(-6.3 - 3.5)	.662	37.4	(32.4 - 42.4)	-7.0	(-13.8 - -0.1)	.062	.212
Obesity	23.3	(20.2 - 26.4)	-0.7	(-5.1 - 3.6)	.750	22.5	(18.2 - 26.8)	-0.4	(-6.5 - 5.6)	.958	.928
Class 1 obesity	16.4	(13.7 - 19.1)	-1.0	(-4.8 - 2.8)	.615	13.3	(9.8 - 16.8)	0.7	(-4.3 - 5.7)	.764	.607
Class 2 obesity	5.4	(3.7 - 7.0)	-0.5	(-2.8 - 1.8)	.694	7.6	(4.9 - 10.3)	-2.8	(-6.3 - 0.7)	.114	.312
Class 3 obesity	1.5	(0.6 - 2.4)	0.7	(-0.7 - 2.1)	.314	1.6	(0.3 - 2.9)	1.7	(-0.5 - 4.0)	.100	.581
Central obesity IDF	68.0	(64.6 - 71.4)	0.7	(-4.1 - 5.5)	.709	62.8	(57.8 - 67.7)	2.8	(-4.2 - 9.8)	.348	.602
Central obesity WHO	42.0	(38.4 - 45.6)	2.4	(-2.7 - 7.6)	.304	34.5	(29.6 - 39.4)	4.1	(-2.9 - 11.2)	.210	.641
**55-64 years**											
Number of subjects	812		746			436		407			
Normal weight	30.5	(27.4 - 33.7)	3.8	(-0.9 - 8.4)	.121	28.9	(24.6 - 33.2)	6.2	(-0.1 - 12.5)	.052	.513
Overweight	39.9	(36.5 - 43.3)	-0.9	(-5.8 - 4.0)	.734	40.4	(35.7 - 45.0)	-4.0	(-10.6 - 2.6)	.240	.454
Obesity	29.6	(26.4 - 32.7)	-2.9	(-7.4 - 1.6)	.214	30.7	(26.4 - 35.1)	-2.2	(-8.4 - 4.0)	.463	.876
Class 1 obesity	18.6	(15.9 - 21.3)	-1.3	(-5.1 - 2.5)	.509	20.9	(17.0 - 24.7)	-3.4	(-8.8 - 1.9)	.199	.530
Class 2 obesity	7.8	(5.9 - 9.6)	-0.5	(-3.1 - 2.1)	.722	7.8	(5.3 - 10.3)	-0.7	(-4.2 - 2.9)	.715	.933
Class 3 obesity	3.2	(2.0 - 4.4)	-1.1	(-2.7 - 0.6)	.192	2.1	(0.7 - 3.4)	1.9	(-0.4 - 4.2)	.116	.044
Central obesity IDF	76.5	(73.6 - 79.4)	2.9	(-1.3 - 7.0)	.166	73.3	(69.2 - 77.5)	2.1	(-3.8 - 8.0)	.496	.757
Central obesity WHO	52.7	(49.3 - 56.2)	-1.8	(-6.8 - 3.1)	.478	48.0	(43.3 - 52.8)	2.3	(-4.5 - 9.1)	.503	.337
**65-74 years**											
Number of subjects	486		715			249		419			
Normal weight	26.3	(22.4 - 30.3)	0.7	(-4.5 - 5.8)	.773	22.5	(17.3 - 27.7)	6.6	(-0.3 - 13.6)	.066	.163
Overweight	40.9	(36.6 - 45.3)	-0.8	(-6.5 - 4.9)	.665	41.4	(35.2 - 47.5)	-2.7	(-10.4 - 5.0)	.484	.744
Obesity	32.7	(28.5 - 36.9)	0.2	(-5.3 - 5.6)	.857	36.1	(30.1 - 42.2)	-3.9	(-11.4 - 3.5)	.319	.341
Class 1 obesity	23.0	(19.3 - 26.8)	0.0	(-4.8 - 4.9)	.880	26.1	(20.6 - 31.6)	-2.7	(-9.5 - 4.0)	.455	.463
Class 2 obesity	6.8	(4.5 - 9.0)	0.1	(-2.8 - 3.0)	.970	8.8	(5.3 - 12.4)	-1.9	(-6.1 - 2.3)	.375	.471
Class 3 obesity	2.9	(1.4 - 4.4)	0.1	(-1.9 - 2.0)	.854	1.2	(-0.2 - 2.6)	0.7	(-1.3 - 2.7)	.502	.573
Central obesity IDF	81.1	(77.6 - 84.6)	-0.8	(-5.4 - 3.7)	.808	77.5	(72.3 - 82.7)	5.1	(-1.1 - 11.3)	.099	.162
Central obesity WHO	58.8	(54.5 - 63.2)	-2.7	(-8.4 - 3.0)	.492	54.2	(48.0 - 60.4)	2.6	(-5.2 - 10.4)	.492	.341
**Total**											
Number of subjects	2023		2174			1054		1184			
Normal weight	33.5	(31.4 - 35.5)	2.1	(-0.7 - 5.0)	.147	31.3	(28.5 - 34.1)	6.7	(2.8 - 10.6)	.001	.071
Overweight	38.5	(36.3 - 40.6)	-1.0	(-4.0 - 1.9)	.491	39.6	(36.6 - 42.5)	-4.6	(-8.6 - -0.5)	.026	.155
Obesity	28.1	(26.1 - 30.0)	-1.1	(-3.8 - 1.6)	.436	29.1	(26.4 - 31.9)	-2.1	(-5.9 - 1.6)	.261	.703
Class 1 obesity	18.9	(17.2 - 20.6)	-0.6	(-2.9 - 1.8)	.642	19.4	(17.1 - 21.8)	-1.8	(-5.0 - 1.5)	.289	.645
Class 2 obesity	6.7	(5.6 - 7.8)	-0.4	(-1.9 - 1.1)	.604	8.0	(6.3 - 9.6)	-1.7	(-3.9 - 0.4)	.110	.326
Class 3 obesity	2.5	(1.8 - 3.2)	-0.1	(-1.1 - 0.8)	.805	1.7	(0.9 - 2.5)	1.4	(0.1 - 2.6)	.038	.080
Central obesity IDF	74.6	(72.7 - 76.5)	1.1	(-1.5 - 3.7)	.398	70.6	(67.9 - 73.4)	3.2	(-0.5 - 6.8)	.090	.342
Central obesity WHO	50.3	(48.2 - 52.5)	-0.4	(-3.4 - 2.6)	.802	44.8	(41.8 - 47.8)	3.0	(-1.1 - 7.1)	.149	.157

*p-value for change, adjusted for age

**Interaction for study area × timepoint, adjusted for age

To examine these findings further, obesity was categorized into subclasses 1, 2 and 3. The prevalence of class 1 obesity decreased in both areas, especially among men in the youngest age group (Table 2). Class 3 (morbid) obesity increased in the control area (from 1.2% to 2.3%, OR 1.97, 95% CI 1.21-3.22, *p = *0.007) and remained unchanged in the FIN-D2D area (from 2.0% to 1.9%, *p = *NS) (Figure 2). The interaction for study area × time point was significant for the genders combined (*p = *0.020), and for the women in the age group of 55-64 years (*p = *0.044). The prevalence of morbid obesity was highest among 55-64 year old women in the control area at the end of the study (4.0%, 95% CI 2.0%-5.8%), and lowest among 65-74 year old men in the control area in the beginning of the study (0.4%, 95% CI -0.4%-1.1%). Altogether, class 2 and 3 obesity were more prevalent among women than among men (6.7% vs. 4.0%, and 2.5% vs. 1.2%, for classes 2 and 3, respectively) (Figure 2).

### Changes in waist circumference and prevalence of central obesity

Baseline waist circumference was larger in women in the FIN-D2D area (89.2 ± 13.2 cm) than in the control area (87.5 ± 12.6 cm) (*p = *0.001). The mean waist circumference increased among women in the control area, but remained unchanged in the FIN-D2D area (Table 1). Here, the interaction for study area × time point for both genders combined was also significant (*p = *0.040). Among men, no significant changes in waist circumference were seen in the control area or in the FIN-D2D area (Table 1). Since the changes in the waist circumference were rather modest, we saw no changes in the prevalence of central obesity in either gender. Central obesity was more prevalent in women than in men (49.3% vs. 36.5%, and 74.5% to 66.8%, WHO and IDF criteria, respectively). The prevalence of central obesity increased with increasing age in both genders (Tables 2 and 3).

## Discussion

The Finnish Programme for the Prevention of Type 2 Diabetes [22] is the first large-scale national programme ever launched to combat the diabetes epidemic at the population-level. The implementation project of this program, FIN-D2D, focused mainly on the high-risk strategy, and the strategy of early diagnosis and treatment [25]. The FIN-D2D also supported the population strategy and it gained a lot of publicity in the whole country because its main aim was to restrain the obesity epidemic by means of nutritional interventions and increased physical activity. As shown in the present study, this goal was achieved, since the mean body weight, mean BMI, and the prevalence of obesity decreased, whereas the prevalence of normal weight increased. This suggests that the prevalence of obesity may be decreasing among 45-74 year old Finns. To our knowledge, this is the first report to show a decline in the prevalence of obesity at the population-level, both in the FIN-D2D and the control area. Interestingly, the waist circumference increased in the control area, whereas it remained unchanged in the FIN-D2D area. However, the increase in waist circumference in the control area was more modest than reported earlier for years 1987-2002 [32], and the prevalence of central obesity did not increase significantly in either area.

There is large geographic variation in the prevalence of obesity among European countries, with central, eastern, and southern regions showing higher rates than the western and northern (including Finland) regions [33]. Studies from Spain, Canada, Denmark, Portugal, and South Australia [34-38] have shown that the prevalence of obesity has increased between the mid-1990s and the beginning of the 2000s. In Finland, national FINRISK studies have shown that the increase in BMI has slowed down in men, and has been quite stable in women between 1997 and 2002, whereas a remarkable shift towards higher waist circumference has been observed [32]. BMI did not increase significantly between 1997 and 2002 in the age groups of 35-64 years, but it increased significantly in the youngest age group, 25-34 years, where also the increase in waist circumference was greatest [32]. Among US adults the increases in the prevalence of obesity previously observed does not appear to be continuing at the same rate during the past 10 years, particularly for women [3]. In Sweden, the increase in the prevalence of obesity has levelled off during 2000-2005 [39] compared to the 10% increase seen during the years 1996-2001 [40]. In our study, a significant decrease in the prevalence of obesity at the population-level was seen for the first time.

In addition to the changes seen in the FIN-D2D area, most changes in body weight and the prevalence of obesity were similar in the control area, especially among men. Although the five hospital districts of FIN-D2D formed a pilot area, the information of the importance of healthy lifestyles in the prevention of diabetes had also spread outside the FIN-D2D area, which was the aim of population strategy, as well. Moreover, the control area is part of the FINRISK area, where the measurements of cardiovascular risk factors have been repeated every five years since 1972 with impressing results [27,28], and they have also received feedback of these results. Therefore, the control area can be considered better as a mini-intervention area rather than a pure control area. The latest FINRISK-survey showed that many cardiovascular risk factors were proceeding into the right direction among 25-64 year old individuals in Finland [4,41]. However, the use of alcohol was still high [42], and this could be a reason why the waist circumference was increasing [32], and why the beneficial trend in blood pressure had now levelled off [4].

There were also substantial differences among the studied areas on how they succeeded in the project, which can largely be explained by their different ways of action in the prevention of chronic diseases, and also their different population structures. It should also be noted that the prevalence of diabetes in the FIN-D2D area has already for a long time been higher than the mean prevalence of diabetes in Finland [25]. The areas were thus establishing this project from different starting points, but they also reached the goals differently. The FIN-D2D was a national health promoting programme, and not an actual scientific study. However, from the very beginning it was decided to analyse its influence by comparing changes in adiposity markers in the FIN-D2D area and the preselected control area.

In this study based on the two cross-sectional surveys with 3 to 5 years of follow-up it was surprising that men in the FIN-D2D area (especially the youngest age group) were able to reduce their body weight more than women. Usually women are more likely to participate in lifestyle change programmes than men [43]. In FIN-D2D there were several projects focusing on men especially, which may have encouraged men to join these projects, and to also succeed in them. The threshold for men to participate is usually higher than that for women, but their commitment nevertheless is usually better [43-45]. Also, the interventions aimed at women may indirectly affect also men due to the fact that men usually eat meals prepared by women [46]. Women in the control area showed beneficial changes in the prevalence of normal weight and overweight, but on the other hand, adverse changes regarding waist circumference and the prevalence of morbid obesity. Of course, one could speculate, whether the result concerning morbid obesity is a real finding or just a statistical coincidence. However, both FIN-D2D and the control population are selected as random independent samples of individuals, specified by region, gender, and age group, being thus a representative sample of the population. Similar changes in the prevalence of morbid obesity were observed in all sub-regions, i.e. the prevalence of morbid obesity increased in all sub-regions of the control area, and remained unchanged, in all FIN-D2D hospital districts.

The prevalence of severe (BMI ≥35 kg/m^2^) and morbid (BMI ≥40 kg/m^2^) obesity has increased in Finland 2.7-fold and 5.6-fold, respectively, during 1978-2001 [47]. Although the number of massively obese individuals is still rather low in Finland, it would be of importance to restrain their number from increasing further. In USA the prevalence of morbid obesity is 5.4% in the age group of 40 or older [3], which is nearly three times the prevalence in Finland (1.9% with FIN-D2D and FINRISK studies combined). Although rather rare, this extreme group of people incur huge expenses along with increasing surgical treatment of obesity.

The present results indicate that the obesity epidemic might be stabilizing or even decreasing in Finland. If this trend is sustainable, also the incidence of type 2 diabetes might follow the obesity trends, and ease off during the years to come. To achieve this goal, we must now be able to maintain and to continue the progression already made regarding body weight. Furthermore, it would also be very important to be able to spread healthier lifestyles into the younger age groups, since obesity appears to reduce life expectancy markedly, especially among younger adults [48].

## Limitations of the study

There were some differences in the age group distribution, since the oldest age group was not included in certain baseline study areas. However, since Turku-Loimaa and Kainuu belong to the control area, and Northern Savo and Northern Ostrobothnia belong to the FIN-D2D area, the age group distribution remains similar between the study groups. These areas represent 39% of the control area population, and 31% of the FIN-D2D area population in the follow-up. In all statistical analysis, age was included as a cofactor. The participation rates were rather low, especially in the control area. We do not know the reason for this. Moreover, there were some differences between the areas concerning certain background factors. Larger urban areas were not evenly distributed between the FIN-D2D and the control area, with the control area including for example the big cities of Helsinki and Turku. Individuals in the FIN-D2D area had slightly shorter duration of education and their cigarette smoking was somewhat less frequent than in the control area, whereas no difference in leisure-time physical activity was seen. When these factors were taken into account, the observed interactions for study area × time point were still significant for waist circumference (*p *= 0.013) and morbid obesity (*p *= 0.028), when both genders were combined. Furthermore, the interaction concerning waist circumference among women became significant with these adjustments (*p *= 0.014), whereas it was not significant without these adjustments (*p *= 0.062). Adding the variable of sub-region into the statistical models had no effect, and was thus left out. Thus, we can conclude that the differences between the areas were not due to these background factors.

## Conclusions

In conclusion, the overall body weight and the prevalence of obesity have been decreasing among Finns aged 45 to 74 years, both at the FIN-D2D and the control area. The significant differences in the prevalence of morbid obesity and mean waist circumference observed between the FIN-D2D area and control area suggest that the implementation of a national diabetes prevention programme may have been successful in the five hospital districts where this programme was established. However, since most of the findings were not significant, we can not be certain of the true extent of the change, and future surveys may give additional data on the direction of this trend in Finland.

## Competing interests

The authors declare that they have no competing interests.

## Authors' contributions

TMS, MP, TS, and MU made substantial contributions to conception, design, and interpretation of the data and they were involved in drafting the manuscript. All authors participated in the design and conduction of the study, acquisition of data and helped to draft the manuscript or revise it. TMS performed the statistical analysis. All authors read and approved the final manuscript.

## Pre-publication history

The pre-publication history for this paper can be accessed here:

http://www.biomedcentral.com/1471-2458/11/350/prepub
